# The Role of Gene Duplication and Unconstrained Selective Pressures in the Melanopsin Gene Family Evolution and Vertebrate Circadian Rhythm Regulation

**DOI:** 10.1371/journal.pone.0052413

**Published:** 2012-12-21

**Authors:** Rui Borges, Warren E. Johnson, Stephen J. O’Brien, Vitor Vasconcelos, Agostinho Antunes

**Affiliations:** 1 CIMAR/CIIMAR, Centro Interdisciplinar de Investigação Marinha e Ambiental, Universidade do Porto, Rua dos Bragas, Porto, Portugal; 2 Departamento de Biologia, Faculdade de Ciências, Universidade do Porto, Rua do Campo Alegre, Porto, Portugal; 3 Laboratory of Genomic Diversity, National Cancer Institute, Frederick, Maryland, United States of America; 4 Theodosius Dobzhansky Center for Genome Bioinformatics, St. Petersburg State University, St. Petersburg, Russia; University of California Santa Barbara, United States of America

## Abstract

Melanopsin is a photosensitive cell protein involved in regulating circadian rhythms and other non-visual responses to light. The melanopsin gene family is represented by two paralogs, *OPN4x* and *OPN4m*, which originated through gene duplication early in the emergence of vertebrates. Here we studied the melanopsin gene family using an integrated gene/protein evolutionary approach, which revealed that the rhabdomeric urbilaterian ancestor had the same amino acid patterns (DRY motif and the Y and E conterions) as extant vertebrate species, suggesting that the mechanism for light detection and regulation is similar to rhabdomeric rhodopsins. Both *OPN4m* and *OPN4x* paralogs are found in vertebrate genomic paralogons, suggesting that they diverged following this duplication event about 600 million years ago, when the complex eye emerged in the vertebrate ancestor. Melanopsins generally evolved under negative selection (ω = 0.171) with some minor episodes of positive selection (proportion of sites = 25%) and functional divergence (θ_I_ = 0.349 and θ_II_ = 0.126). The OPN4m and OPN4x melanopsin paralogs show evidence of spectral divergence at sites likely involved in melanopsin light absorbance (200F, 273S and 276A). Also, following the teleost lineage-specific whole genome duplication (3R) that prompted the teleost fish radiation, type I divergence (θ_I_ = 0.181) and positive selection (affecting 11% of sites) contributed to amino acid variability that we related with the photo-activation stability of melanopsin. The melanopsin intracellular regions had unexpectedly high variability in their coupling specificity of G-proteins and we propose that Gq/11 and Gi/o are the two G-proteins most-likely to mediate the melanopsin phototransduction pathway. The selection signatures were mainly observed on retinal-related sites and the third and second intracellular loops, demonstrating the physiological plasticity of the melanopsin protein group. Our results provide new insights on the phototransduction process and additional tools for disentangling and understanding the links between melanopsin gene evolution and the specializations observed in vertebrates, especially in teleost fish.

## Introduction

Vertebrates have a wide range of strategies to respond to light in different photic environments [1]. The evolution of these diverse light-signalling processes and the link between photoreceptors and adaptive strategies are not fully understood. One of the most-recently discovered groups of photoreceptors, melanopsin (*OPN4*), was first described in the dermal melanophores of *Xenopus laevis* [2]. Its main functions are non-image forming, including the regulation of circadian rhythms, the pupillary light reflex and melatonin synthesis [3]–[5]. Melanopsins are sensitive to low wavelength light with maximum sensitivities near to 480 *nm* [6], [7].

Within vertebrate genomes there are two variants of the melanopsin gene: the mammalian-like melanopsin (*OPN4m*) and the *Xenopus*-like melanopsin (*OPN4x*) [8]. In mammals, only the *OPN4m* gene has been described, suggesting that the *OPN4x* variant was lost during mammalian evolution [9]. Mammalian melanopsin is expressed in a subset of intrinsically photosensitive retinal ganglion cells (ipRGCs) of the eye [10] while the non-mammalian vertebrates also express melanopsin in intraocular photoreceptors such as the pineal gland and deep brain [11], [12]. Recently, numerous melanopsins were describe in teleost fish including *OPN4x1*, *OPN4x2*, *OPN4m1*, *OPN4m2* and *OPN4m3* [13].

Melanopsins are members of the G protein-coupled receptor (GPCR) protein family that is characterized by a heptahelical transmembrane conserved structure and the activation of a G-protein in their signalling transduction pathway [14]. Melanopsin structure includes seven helical transmembrane domains (TD), three intracellular (IL) and three extracellular (EL) loops, eight cytoplasmic domain (CD8), and N and C-terminals [15]. Residues that are critical for correct melanopsin conformation include: (i) two cysteine residues in the TD3 and EL2 domains that are involved in disulfide bond formation, (ii) a tyrosine and a glutamic acid in the TD3 and EL3 domains, respectively, that act as counter ions to the positive charge of the protonated Schiff base, (iii) a DRY motif at the TD3/EL2 boundary that provides a negative charge to stabilize the inactive opsin molecule, (iv) a lysine residue in the TD7 domain that is covalently linked to the retinal chromophore, and (v) a conserved NPxxY(x)2,3HPKF (NP-Y-F) motif in the TD7-CD8 region conferring structural integrity upon pigment activation [15], [16].

Koyanagi et al. proposed that rhabdomeric opsins evolved in protostomes to provide visual functions (*InRHO*) and in deuterostomes to provide non-visual functions (*OPN4*) [17]. It is hypothesized that all rhabdomeric photoreceptor share the same signal transduction pathway, including the activation of phospholipase C (PLC) and the inositol phosphate (IP3) pathway, which involves the Gq/11 G-protein type [18]. There are three families that constitute the major functional classes of G proteins and that are likely to mediate the melanopsin phototransduction cascade. The Gs and the Gi/o classes of G-proteins mediate the opposing effects of stimulation and inhibition of adenylate cyclase activity, and the Gq/11 family activates phospholipase C enzymes, resulting in phosphatilinositol hydrolysis [19]. Recently, a Gq11-triggered PLC light-signalling cascade was described in amphioxus [20], but a general model for vertebrate melanopsin phototransduction pathway is still missing. However, expression patterns in heterologously [18], [21] and cultured melanophores and ipRGCs cells [22], [23] strongly suggest the involvement of a Gq–based pathway.

Since the regulation of phototransduction in vertebrates is a very complex task, the study of the melanopsin gene family would increase our understanding of the evolution of vertebrate circadian rhythm regulation and would provide insights on the molecular-based adaptations of photoreception during vertebrate evolution. The goal of this study was to assess the selection patterns and evolutionary history of the melanopsin (*OPN4m* and *OPN4x*) paralogs at the gene and protein level. We tested the role of gene duplication and non-synonymous positively-selected substitutions in producing the extant diversity of physiological responses of melanopsin in both visual and non-visual photoreception organs and assessed the selective pressures on the retinal-related sites that determine the spectral absorption of melanopsins and the IL3 and IL2 that are involved in signalling light at the intracellular level. We also described the lineage-specific duplication that occurred in teleost fish that conferred novel photic capacities in new photic environments. Finally, we investigated the physiological plasticity of melanopsins by inferring the G-protein coupling proclivities of each gene.

## Results

### The Evolutionary History of Melanopsins

To understand the origin of melanopsin protein family, 51 *OPN4* gene sequences were retrieved from the Ensembl and NCBI databases from the main groups exhibiting melanopsins, including echinoderms and chordates (**Table S1**). The sequences were obtained by blasting both annotated-sequence databases and non-annotated genomes. To describe the emergence of melanopsin we compared available rhabdomeric photoreceptor sequences, including both melanopsin and invertebrate rhodopsin genes. Rhabdomeric photoreceptors comprehend two distinct evolutionary lineages: the *InRHO* that are present in protostomes, and the *OPN4* from deuterostomes [24]. Although our phylogenetic analyses support this partitioning, we found that the echinoderms comprise the basal branch for rhabdomeric photoreceptors. However, we cannot determine at this time whether it is a true member of the melanopsin gene family or perhaps another rhabdomeric photoreceptor type that has not yet been described. Moreover, rhabdomeric photoreceptors showed a considerable degree of amino acid variability (0.307±0.027 in *InRHO*, 0.580±0.014 in *OPN4x* and 0.614±0.021 in *OPN4m*) relative to their ciliary relatives (0.175±0.020 in *RHO*).

**Figure 1:**
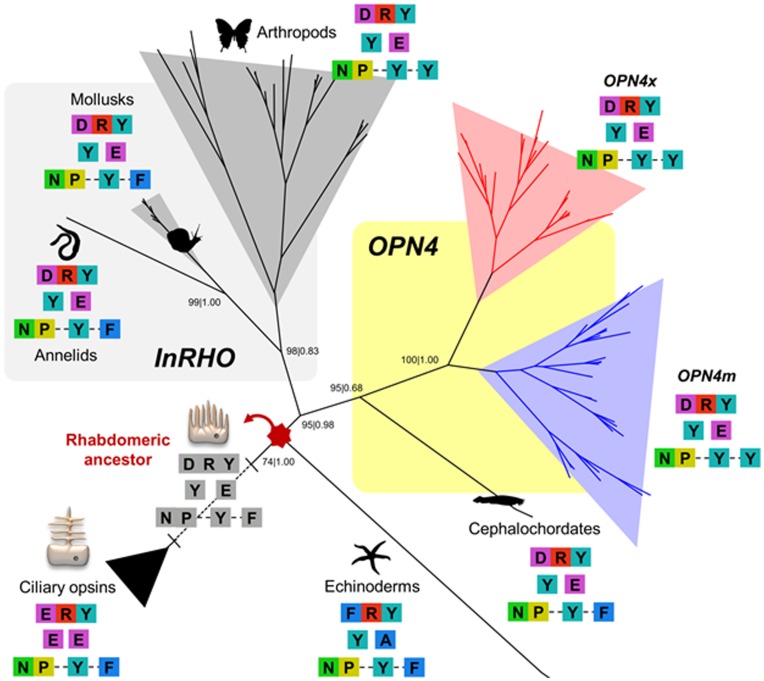
Phylogenetic depiction of the common-ancestry of invertebrate rhodopsins (*InRHO*) and melanopsin. The main opsin amino acid substitutions which are critical for the protein functional and structural innovations are color-coded. Maximum likelihood (ML) and Bayesian methods were used to build the phylogenetic tree and the support values of each method are shown for the main nodes (bootstrap and posterior probability, respectively). The grey amino acids are the maximum likelihood predicted motifs of the rhabdomeric photoreceptor ancestor.

There were several amino acid patterns that broadly track opsin function and structure during rhabdomeric photoreceptor evolution (**figure 1**). Notably, echinoderms presented a FRY motif instead of the characteristic DRY motif of the rhabdomeric family, the E counterion found in all rhabdomeric opsins is replaced by an A in echinoderms and the stability residues of the CD8 domain had an analogous substitution in arthropods and vertebrates (F→Y). Furthermore, we inferred the maximum-likelihood ancestral sequence of the rhabdomeric ancestor and the most-likely ancestral characters of the DRY, Y and E counterions and the NP-Y-F motifs. Remarkably, these are the same amino acid motifs found in the rhabdomeric photoreceptors of extant annelids, mollusks and cephalochordates.

**Figure 2:**
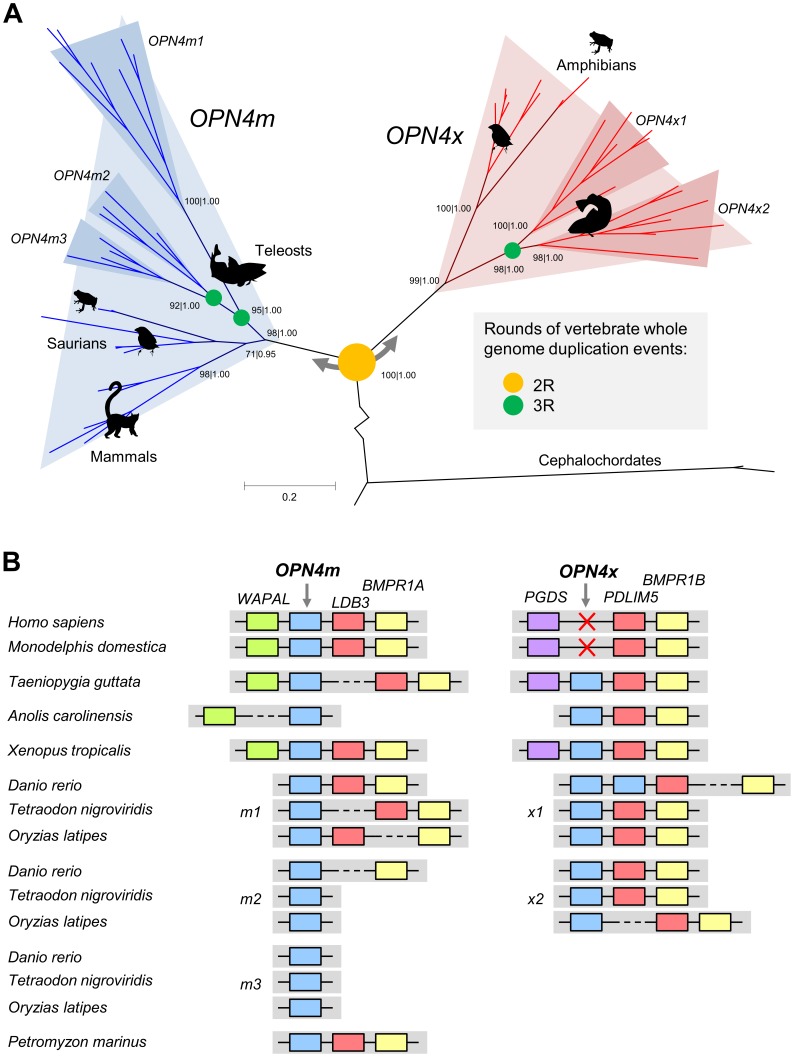
Melanopsin gene tree and the syntenic analyses in the melanopsin genomic paralogon. A. The phylogenetic analyses were retrieved with maximum likelihood and Bayesian methods and the support values for each method (bootstrap and posterior probability, respectively) are shown on the main nodes. The main duplication events that characterize melanopsin gene history are represented with yellow (2R) or green (3R) circles on the respective nodes. B. Paralogous genes are represented with the same color code (*LDB3*/*PDLIM5* and *BMPR1A*/*BMPR1B*). The red cross represents the gene loss in the mammalian *OPN4x*.

Despite the fact that we found melanopsin representatives in cephalochordates and vertebrates, BLAST searches of the available urochordate (*Ciona intestinalis* and *C. savignyi*) genomes, nucleotide collections and expression sequence-tag libraries were inconclusive (no sequence matches were retrieved with a high similarity level). The phylogenetic tree of vertebrate melanopsins (**figure 2A**) highlighted melanopsin evolutionary history, which included the duplication events leading to the origin of the *OPN4m* and *OPN4x* paralogs (2R, second round of whole genome duplication) and the teleost fish duplications leading to *OPN4m1*, *OPN4m2*, *OPN4m3*, *OPN4x1* and *OPN4x2* (3R, third round of whole genome duplication) [8], [13]. These nodes are supported by high bootstrap and posterior probability values (higher than 95 and 0.95, respectively).

Although we did not find a complete sequence of either *OPN4m* or *OPN4x* in the lamprey (*Petromyzon marinus*), our blast searches identified an incomplete DNA fragment (ENSPMAG00000006406) that resembled an *OPN4m* melanopsin variant and phylogenetic analyses grouped the sequence with the *OPN4m* clade with 94% bootstrap and a posterior probability of 1.00 (**figure S1**). Since lampreys are one of the basal groups of vertebrates, this suggested that the melanopsin duplication event occurred earlier, before the emergence of cyclostomes. Also, our synteny analyses showed that the lamprey OPN4 genomic neighborhood includes the *LDB3* gene, which is congruent with observed patterns in the *m*-type paralog found in all other vertebrate taxa (**figure 2B**).

In the monotreme platypus (*Ornithorhynchus anatinus*) genome, our blast searches only found evidence of the *OPN4m* (ENSOANG00000010446) variant, indicating that the *OPN4x* variant was lost early in mammalian evolution, corroborating previous findings that suggested the absence of the gene in the marsupial *Sminthopsis crassicaudata* and placental mammals [11]. Therefore, the *PGDS-OPN4x-PDLIM5* paralogon found in all tetrapoda, could be different in mammals because *OPN4x* was lost earlier in the mammalian ancestor (**figure 2B**). This hypothesis is supported by: (i) our synteny analyses that showed the absence of the *OPN4x* gene in the genomic segment between the *PGDS* and *PDLIM5* genes in all mammals (monotremes, methatheria and eutheria), (ii) by our blast searches in mammals that did not retrieve any matches with the OPN4x protein and (iii) because the *OPN4x* transcript was missing in the very exhaustive human and rat expressing sequence tags databases.

The Onho hypothesis advocates that two rounds of whole genome duplication occurred between the origin of chordates and the origin of jawed vertebrates, likely explaining the great number of paralogous genes in vertebrate genomes [25]. The existence of the *OPN4m* and *OPN4x* paralogs in vertebrate genomes, in addition to our evidence of the *m*-paralog in the lamprey genome, is consistent with the 2R event (**figure 2B**).

Whole genome duplication events shaping the genomes of vertebrates have not only been proposed in the early evolution of vertebrates, but also in the stem lineage of teleost fish, after their divergence from the land vertebrates (3R) [26]. We advanced that the melanopsin lineage specific duplications found in teleost fish (*OPN4m1*, *OPN4m2*, *OPN4m3*, *OPN4x1* and *OPN4x2*) probably occurred around 320 mya (3R event, **figure 2B**) [27], [28].

### Selective Pressure and Conservation in Melanopsins

Evidence of positive or negative selection at specific amino acid residues in vertebrate melanopsins was assessed based on the ratio of nonsynonymous (*d*N) versus synonymous (*d*S) substitutions (*d*N/*d*S or ω). A ω value less than 1 is indicative of purifying selection acting against amino acid changes, whereas a ω value greater than 1 suggests an excess of amino acid changes, indicative of adaptive evolution [29]. To test for positive selection at individual nucleic acid codons we used the site-specific models implemented in codeml program of PAML v4 package [30].

There was no evidence of significant positive selection at the nucleotide site level in *OPN4m* or *OPN4x* under model M8 of positive selection. Similarly, the global ω value under model M7 of no positive selection was very low in both cases (0.172 in *OPN4m* and 0.170 in *OPN4x*, **table 1**) indicating that the evolution of melanopsins in vertebrates was constrained by very stringent selective pressure. Our results contrasted the analyses performed by Dong *et al.* (2010), which reported a 0.07 global omega value for melanopsins [31], largely because we performed separate tests for each melanopsin paralog, used fewer mammalian sequences to reduce saturation bias in our alignments and because we implemented the more-appropriate M7–M8 test-comparison to infer negative selection instead of M8a-M8. The neutral M8a model implements an omega value that is fixed and equal to 1 [32], allowing the discrimination between neutral or positive selection.

**Table 1:** Site-specific selection models for the vertebrate melanopsin *OPN4m* and *OPN4x* genes.

Gene	Model	ω	*lnL*	Hyphothesis	LRT	*df*	
*OPN4m*	A. M0	.132	–17125.136				
	B. M3	.170	–16379.268	A *vs.* B	1491.736	4	*
	C. M7	.172	–16377.538				
	D. M8	.172	–16377.500	C *vs.* D	0.077	2	
*OPN4x*	A. M0	.127	–13962.083				
	B. M3	.168	–13413.817	A *vs.* B	1102.532	4	*
	C. M7	.170	–13408.551				
	D. M8	.177	–13406.677	C *vs.* D	3.748	2	

The likelihood values and the respective estimated parameters are shown for each model. The ω ratio is an average over all sites of the *OPN4m* and *OPN4x* paralogs. The asterisk (*) means that the alternative hypothesis is statistically significant at a 5% level, implementing the LRT (likelihood ratio test). Notes: *df* – degrees of freedom.

**Figure 3:**
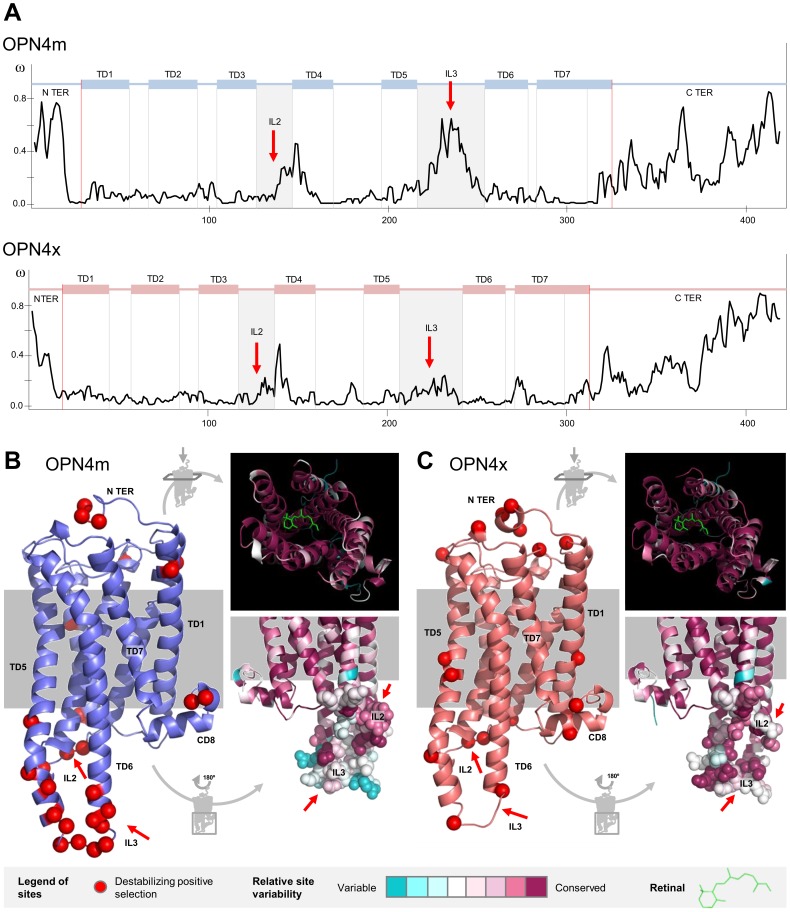
Destabilizing positively selected sites and conservation index in the *OPN4m* and *OPN4x* paralogs. A. ω-ratio site estimation for each melanopsin paralog. The IL2 and IL3 regions are highlighted (red arrows). **B and C.** Three-dimensional structure of *OPN4m* and *OPN4x* paralogs showing the sites under positive destabilizing selection (red) and detailed perspectives of the conservation index in the interior of the molecule, where the retinal is accommodated, and the IL2 and IL3 loops (red arrows).

To further assess selective pressure among sites and to characterize the slow- or fast-evolving domains of melanopsins, we plotted the variation of the ω value for the *OPN4m* and *OPN4x* codon-sites (**figure 3A**). This demonstrated that despite the strong purifying selection experienced by the *OPN4x* and *OPN4m* paralogs, some regions of the molecules accumulated non-synonymous variation. To avoid overestimating the ω value on the N terminus, since some sites are not fully represented for all taxa, we excluded the final part of the N terminus on the **figure 3A** diagrams.

The Mann-Whitney test was used to calculate W statistics [33] and to test the alternative hypothesis of significantly greater median-ω-values in the suspected regions. We tested the ranks of the suspected sites (n) against the remaining sites (N – n) using the same total number of sites for each paralog (N = 420). The C and N-terminus melanopsin domains evolved at higher ω-values (W = 30304*, n = 123 in *OPN4m* and W = 33375*, n = 126 in *OPN4x*), suggesting more amino acid variability in terminal regions. Also, a higher ω value was observed in the second and the third intracellular loops (IL2 and IL3) as well as the helix bundles that comprise each loop (W = 13541.5*, n = 58 in *OPN4m* and W = 13109.5*, n = 63 in *OPN4x*). Together, these regions (plus the CD8 domain) interact with the G-protein that mediates the phototransduction pathway [34].

Further insights on the relationship between melanopsin structure and function were obtained through a protein-level approach by combining information from the three-dimensional melanopsin structure and the physico-chemical properties of the amino acid substitutions. TreeSAAP v3.2 was used to reconstruct ancestral sequences and to determine and categorize evolutionary changes in 30 amino acid properties [35]. We looked for positively selected sites under destabilizing selection (non-synonymous substitution with significant disequilibrium changes to the molecule) and found that 70% of the substitutions had probable chemical implications and 30% had structural implications in both paralogs (**Table S2 and Figure S2**). As expected, substitutions that potentially changed chemical properties were more common than substitutions with structural implications. Thus the heptahelical conformation of melanopsins was safeguarded throughout evolution.

27 and 21 sites were under destabilizing positive selection in both OPN4m and OPN4x, respectively (**figures 3B and 3C** **)**. A chi-square adjustment test with a 5% level cutoff showed that destabilizing positive selected sites had a differential distribution between the extra and intra-membrane regions of the protein (χ^2^ = 10.703* in OPN4m and χ^2^ = 5.762* in OPN4x, both tested at 1 degree of freedom). A large proportion of sites under destabilizing positive selection were located in the IL2 and IL3 and in the helix bundles that comprise each loop (**figures 3B and 3C**). This pattern is more evident in OPN4m (15/27 = 0.56) than in OPN4x (5/21 = 0.24). The predicted three-dimensional conformation of melanopsin showed that these specific sites are located on the intracellular part of the molecule where the G-protein interaction is established. As in the results obtained in the site selection analysis, the conservation index estimated on the Consurf webserver [36] showed that (i) both the N and C terminus are highly variable, (ii) the second and third intracellular loops are unexpectedly variable and (iii) the molecule interior, responsible for the retinal accommodation, is very conserved (see detailed aspects in **figures 3B and 3C**). The proportion of variable sites on the melanopsin molecule was around 55% in *OPN4m* and 59% in *OPN4x*.

### OPN4 Duplications and Functional Divergence

Melanopsin evolutionary history has been marked by a series of gene duplications episodes (**figure 2**). Therefore, we tested for branch and branch-site selection for the main duplication events of melanopsins (*OPN4m*/*OPN4x*, *OPN4m3*/*OPN4ma* and *OPN4x1*/*OPN4x2*). In addition, we assessed the type I and type II functional divergence between variants using Diverge v2.0 [37]. Type I functional divergences represent amino acid configurations that are highly conserved in one clade, but are variable in the other clade, denoting residues that have experienced differentiated functional constraints at a particular site. Type II represent residues which are very different between clades, but are found in very conserved amino acid configurations in both clades, implying that these residues may be responsible for functional specification, especially when the substitution has some biochemical significance [38]. Type I and type II functional divergence tests for each group of duplicates are summarized in **table 2** and the additional information on the branch and branch-sites tests, the estimated parameters and the inferred selected amino acid sites are presented in **table S3**. All the numerical and amino acid identification of sites are based on the *Gallus gallus* OPN4m and OPN4x protein sequences.

**Table 2:** Type I and type II divergence between the OPN4 paralogs and the teleost lineage-specific duplications.

	*OPN4x*/*OPN4m*	*OPN4x2*/*OPN4x1*	*OPN4m3*/*OPN4ma*
Residues	294	339	330
θ_I_ ± *se*	0.349±0.059*	0.039±0.082	0.181±0.082*
*z*_I_	6.362	0.712	3.336
*p*–value	0.000	0.238	0.000
θ_II_ ± *se*	0.126±0.084*	0.044±0.058	0.048±0.061
*z*_II_	2.166	0.799	0.874
*p*–value	0.016	0.212	0.191

θ_I_ and θ_II_ are the coefficients of type I and II functional divergence. Asterisks (*) mark results with statistical significance at 5% level of confidence and *se* denotes the standard error.

**Figure 4:**
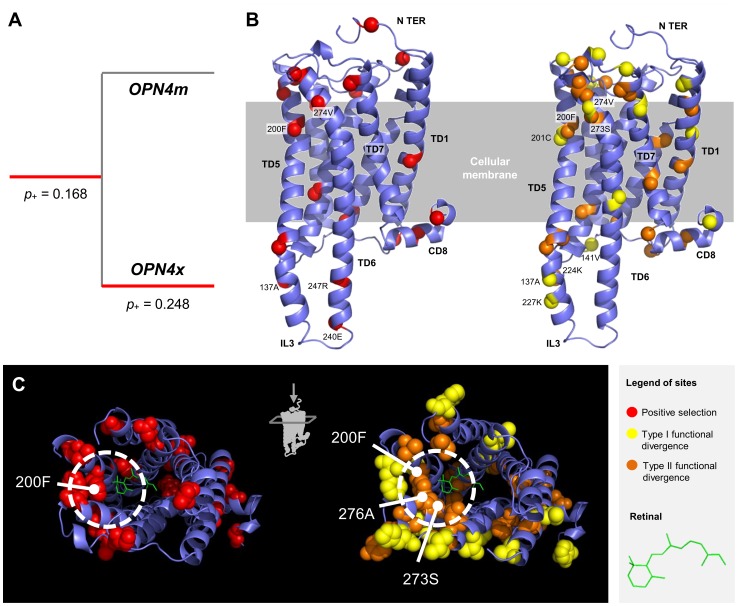
Branch and site selective pressures during the *OPN4m/OPN4x* duplication event. A. Branch-site tests. Red lineages represent an inferred episode of positive selection. In those branches is represented the *p+* parameter (proportion of the positively selected sites). **B.** Representation of the positively selected and functional divergence sites (type I in yellow and type II in orange) in the three-dimensional structure of the *Gallus gallus* OPN4m protein. **C.** A detailed perspective of the retinal accommodation on the melanopsin molecule and the occurrence of the positively selected and type I and II functional divergence sites.

After the *OPN4x/OPN4m* duplication event, the number of non-synonymous substitutions increased which led to a higher overall ω-ratio on these lineages. 25% of the melanopsin sites were under positive selection in the *OPN4x* lineage. There was a significant functional divergence between *m*-melanopsin and *x*-melanopsin, indicated by 6% and 8% of the sites being under type I and type II functional divergence, respectively. Positively selected sites, as those involved in type I and type II functional divergence on the *G. gallus OPN4m* three-dimensional structure are displayed graphically in **Figure 4**. A group of residues on the initial regions of the TD5 and TD4 (200F, 273S and 276A) that are involved in retinal connection showed evidence of functional divergence and/or positive selection (**figure 4C**). The IL3 and IL2 and the respective bundles both had sites with signals of positive selection or that contributed to functional divergence (e.g. 137A, 141V, 224K, 227K, 240E and 247R).

At least two whole duplication events were fundamental in determining actual teleost *m*-type melanopsin patterns (*OPN4m3/*[*OPN4m2+OPN4m1*], *OPN4m1/OPN4m2*), but only one even is sufficient to explain *x*-type evolution (*OPN4x1/OPN4x2*). To simplify the clade notation, when refer to *OPN4m2*+ *OPN4m1* clade instead as *OPN4ma*. Taking into account both phylogenetic and synteny analyses in teleost fish, we studied the three duplication events (**figure 2A**) of teleost melanopsin paralogs in more detail. Due to an insufficient amount of available sequences for the *OPN4m1* and *OPN4m2* duplicates, we have not done a branch-site or functional-divergence analysis for the *OPN4m1/OPN4m2* duplication event.

**Figure 5:**
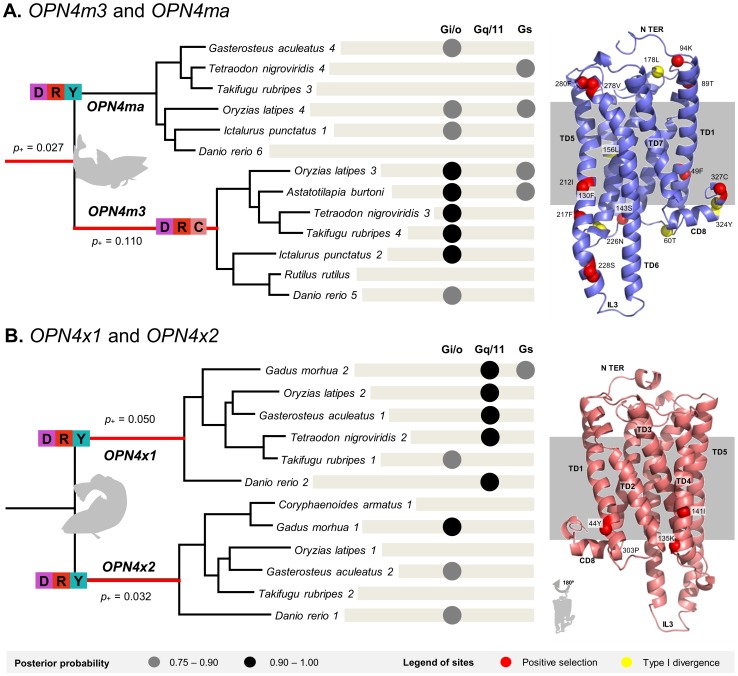
Branch and site selective pressures during the teleost lineage-specific duplications: A. *OPN4m3/OPN4ma* and B. *OPN4x1/OPN4x2*. A punctual substitution (Y→C) was determined in the DRY motif in the OPN4m3 teleost melanopsin duplicant. Red lineages represent an episode of positive selection and the *p*+ parameter means the proportion of the positively selected sites. Black and grey circles represent the posterior probability level of G-protein coupling preference for each teleost fish amino acid sequence: 0.75–0.90 (grey circles) and >0.90 (black circles). The three-dimensional structure of the *Gallus gallus* OPN4m and OPN4x paralogs is also represented showing the occurrence of positive selection and functional divergence at the site level.

In the *OPN4m3* lineage 11% of the residues were under positive selection and both copies showed evidence of type I but not type II functional divergence. The main residues responsible for positive selection and functional divergence are located in the TD5 and the CD8 regions (**figure 5**). Moreover, we found that *OPN4m3* protein sequences of the DRY were replaced by the DRC motif. Both lineages of the *OPN4x1*/*OPN4x2* duplication were under positive selection, although to a lesser extent (around 5%), and no evidence of functional divergence was found between these copies.

### G-protein Couple Receptors

Melanopsins process light by using a G-protein that establishes a physical-chemical interaction with the intracellular domains of the opsin. We used Pred-Couple v2.0 web server to determine the potential G-protein couple preferences of GPCRs on the four possible subfamilies (Gs, Gi/o, Gq/11 and G12/13) [39]. We found that melanopsins have a possible promiscuous interaction with two G-proteins: Gi/o and Gq/11. There was no evidence that G12/13 was a coupling G-protein, which increased confidence in the accuracy of our results, as this is a ciliary-type G-protein.

For the teleost fish melanopsin duplications, the *OPN4x1* copy showed affinity with the Gq/11-type and *OPN4m3* with the Gi/o, both with >0.90 posterior probability level (**figure 5**). Both x-type and m-type melanopsins in birds had affinity with the Gq/11 G-protein (0.89 and 0.84 in *OPN4m* and *OPN4x* on *Gallus gallus* amino acid sequences). In mammals, higher affinity was also observed for the Gq/11-type G-protein with a posterior probability of 0.96 and 0.91 in *Canis familiaris* (Laurasiatheria representative) and *Loxodonta africana* (Afrotheria representative), respectively. Therefore, Gq/11 was the most likely G-protein intervenient in the melanopsin phototransduction cascade, especially in non-fish vertebrates.

## Discussion

Understanding the molecular evolution of photoreceptor genes is crucial to assessing how genetic variation influences molecular specialization and to understanding the implications to how organisms have adapted to different photic environments. At the molecular level melanopsins may have specialized by (i) establishing distinct coupling preferences with the signalling cascade in the cell interior and/or (ii) changing their spectral sensibility accordingly to environmental conditions. The implications of which are discussed below.

### Integration of Light by Melanopsin – the Variability of the Second and Third Intracellular Loops (IL2 and IL3) and G-protein Type Preferences

Our evolutionary analyses of the rhabdomeric photoreceptors suggest an urbilaterian common-ancestor for both *OPN4* and *InRHO* orthologs (**figure 1**). This result corroborates the general Arendt theory of photoreceptor cell-type evolution [24], [40] that supposes a rhabdomeric-like cell in the set of photoreceptors of the ancient urbilaterian eye. Additionally, the inferred ancestral amino acid sequence for the urbilaterian rhabdomeric ancestral photoreceptors suggests that the molecular basis of rhabdomeric-like light transduction remained similar to that observed now. Therefore, some extant groups (annelids, mollusks and cephalochordates) have the same combination of amino acid motifs (**figure 1**). This result supports the idea of a universal method of signalling light in the rhabdomeric photoreceptors, at least in the mechanisms of retinal biding and structural maintenance that these amino acid motifs perform.

Furthermore, experimental studies show that all rhabdomeric photoreceptors share the same signal transduction pathway, including the activation of the phospholipase C (PLC) and the inositol phosphate (IP3), which involves the Gq/11 G-protein type [17], [18], [20], [41]. However, we determined that there is possible uncertainty in the affinity of teleost fish melanopsins relative to their G-protein couple preferences: Gi/o and Gq/11 (**figure 5**). It should be stressed that for the mammals and birds studied here, the Gq/11 was always predicted to be the most-likely intervening G-protein type. We propose that these promiscuous coupling preferences in teleost fish may constitute an evolutionary advantage since one environmental signal may produce a great quantity of internal organism responses. We suggest that this behavior may provide an ecological advantage by originating new and more complex photo-irritability responses to environmental stimuli. Moreover, we observed unexpected variability in the IL2 and IL3 loops suggesting, in agreement with the previously- discussed result, the ambiguous activation of more than one G-protein. We advance three possible resolutions to this quandary: (i) Gq/11-type G-proteins do not require conserved intracellular domains to establish a coupling ligation in melanopsins, (ii) intracellular loop variability contributes to G-protein coupling promiscuity on melanopsins, or, a less-likely but possible explanation that (iii) another type of G-protein mediates the melanopsin phototransduction pathway.

### *OPN4m* and *OPN4x* Paralogs and the Emergence of the Complex Eye in Vertebrates

We found that melanopsins were apparently lost in tunicates, whereas only one copy is present in cephalochordates and vertebrates present two copies. Gene loss in urochordates is generally assumed to be common, and it was already reported for the well-studied *Hox* genes [42], [43] so we hypothesize that melanopsin may have been lost during a genomic rearrangement process. However, regardless of the quality of the genome assembly, it should be noted that negative results from gene searches in genomes or DNA libraries may be biased because of incomplete genome sequence, the lack of protein homology or missing sequence data. To date, the *Ci-opsin1* and the *Ci-opsin2* ciliary opsin genes involved in photic stimuli in larval stages have been identified in *C. intestinalis,* but other types of photoreceptors cells have also been identified [44], [45]. More molecular studies are needed to more-thoroughly evaluate the presence or absence of a rhabdomeric-like photoreceptor in urochordates genomes, which would be of great importance in disentangling the ancestral photoreceptor content of the vertebrate eye.

All vertebrates have anatomical features that are not observed in their closest living relatives, the urochordates and cephalochordates. It has been shown that the 1R and 2R whole genome duplication events seem to explain the photomorphological diversity that we can currently see in vertebrates [46]. Cyclostomes are a very basal group in vertebrate phylogeny and the presence of the *OPN4m* variant introduced by us (**figure S1**) is consistent with a whole genome duplication event just before the emergence of jawless fish, coincident with the 2R episode. Moreover, the presence of genomic paralogons among vision-related genes produced by the 2R episode seems to be common pattern in visual opsins, as has been demonstrated through the study of the protein intervenes in the vertebrate visual cascade [47]. The syntenic and phylogenetic analyses of *OPN4m* and *OPN4x* (**figure 2**) suggest that a whole genome duplication event occurred during the emergence of vertebrates, as with the 2R episode. These result predicts that the emergence of melanopsin variants parallel the vertebrate emergence (at least 600 mya), earlier than the origin of the Tetrapoda in the Late Devonian (360 mya) as proposed by Bellingham et al. (2006) [8].

However, the question remains as to why both paralogs were maintained in the genome following the duplication event. We hypothesize that an advantageous dosage effect can explain the retention of the duplicated melanopsin paralogs in the genome [48], [49]. We assume that a photoreceptor dosage effect could have been be of great advantage, or at least more advantageous than the expected metabolic constraints such as energy loss and the regulation of the signalization pathways. Not only the organization of the non-visual system went through dramatic changes during the emergence of vertebrates, but the visual system also changed significantly, as demonstrated by the photoreceptors and their current paralogs (e.g. rhodopsins and conopsins), and as such are arguably the principle reason for the development of complex eye novelty [50]–[52]. Thus, the complex visual system of vertebrates is the result of the large number of photoreceptors that enable the processing of wavelengths in different ranges of the light spectra (visible and also UV). The melanopsin group, as well as the ciliary opsins (e.g. rhodopsins and conopsins), show diverse duplicated copies (*Rh1*, *Rh2*, *SWS1*, *SWS2* and *LWS*) that over time underwent further specialization, and which presently regulate important processes such as color-vision or circadian-rhythm synchronization [53].

### Melanopsin and Site Level Selective Pressures – Evidence of Spectral Sensibility Specialization

Our results show that melanopsin amino acid substitutions are mainly under negative selection. This suggests that melanopsins play an important physiological role in the photoreception system and that the complete or partial loss of melanopsin functionality would compromise organism fitness. Indeed, mammalian melanopsin is responsible for phase-shifting circadian rhythms, plasma melatonin suppression, spanning pupil constriction and the dependent irradiance regulation of retinal cone function [54]. These functions are related with basic physiological needs, such as feeding and reproduction, thus justifying the need of fine-scaled regulation at the genetic level. Among non-mammalian vertebrates, several photoreceptive locations have been well-described in addition to the retina, including the pineal gland and deep brain [55], [56]. In these extra-retinal photoreceptors, the role of melanopsin is not completely understood, and since both melanopsin paralogs are present in non-mammalian vertebrates, inferences of selective pressure acting in these lineages should be made with caution. Despite the indication of a general purifying selective signature mediating melanopsin evolution, we identified several sites that are responsible for both selective and functional divergence between the *m* and *x* melanopsins. The *OPN4x* lineage showed evidence of positive selection, which suggests a relaxation of the selective pressure favoring genetic variation following the post-duplication episode. Additionally, functional divergence types I and II were detected, indicating a process of functional differentiation and specialization over 600 mya of vertebrate evolution. Indeed, we show that some sites under positive selection and functional divergence near the retinal localization (200F, 273S and 276A) (**figure 4**) with likely implications to spectral sensibility. It has been shown that in cones and rhodopsins the sites responsible for spectral tuning tend to cluster around either the Schift base linkage or the ionone ring of retinal [57]. In contrast, in chicken (*G. gallus*) the m and x-type melanopsins showed the same spectral sensibility (476–484 nm) [58]. However, zebra fish spectral sensitivity for *OPN4m3* and *OPN4x2* is highest at 484 *nm* and 470 *nm*, respectively [13].

### The 3R Event and the Large Number of Melanopsin Paralogs in the Teleost Eye

We hypothesize that melanopsin copies may have been key to the radiation of teleost fish (3R event, **figure 2B**), playing a major role by providing new photic capacities in new environments. Aquatic environments are very complex from the photic point of view, varying based on numerous factors including turbidity, salinity, pressure and depth that result in very different refractive indexes throughout the water column [59]. Thus, the existence of many photoreceptors would be an advantage in such complex ecosystems. Interestingly, we identified five melanopsin representatives in the teleost retina while most of vertebrates have two, implying the existence of a complex non-visual signalling pathway in teleost fish and the involvement of multiple protein complexes.

Moreover, it is known that *OPN4m3*, *OPN4x1* and *OPN4x2* are monostable photopigments, while instead *OPN4m1* and *OPN4m2* display invertebrate-like bistability [13]. Bistable pigments are thermally stable before and after photo-activation, but monostable pigments are stable only before activation [60]. Accordingly, our results suggest that a process of functional divergence and diversifying positive selection occurred on the *OPN4ma* (*OPN4m1*+ *OPN4m2*) and that *OPN4m3* is located mostly on the TD5 and CD8 domains (130F, 156L, 178L and 324Y) (**figure 5**). These domains may play an important role conferring structural ability to these pigments to perform the monostability or bistatibilty types of retinal accommodation. Indeed, CD8 domain is known to be involved in conferring structural integrity upon pigment activation [61]. Furthermore, OPN4m3 protein presents a substitution on the DRC motif, which may have implications to provide the negative charge to stabilize the inactive opsin. For the *x*-type duplications, we did not find any type of functional divergence between *OPN4x1* and *OPN4x2*, which are both monostable photopigments.

### Conclusions

Our general results suggest that the main phenomena determining melanopsin gene family evolution are (1) purifying negative selection and (2) the duplication events followed by minor episodes of positive selection and functional divergence.Negative selective pressures help maintain the structural and biochemical homology observed among all opsin photoreceptors and duplication events are the source of gene number variation in the vertebrate genomes. In addition, the variability at the amino acid level is mostly located at the retinal biding-related sites and in the third and second intracellular loops. This suggests that vertebrate melanopsin adapted to new photic environments by one or both of these processes: providing sensibility to different quality and quantity of light and/or supplying new or more complex photo-irritability responses.

## Methods

### Data Collection

PSI-BLAST and TBLASTN searches with protein sequences of the two *Gallus gallus* melanopsins (NM_001044653.1 and AB255031.1) were performed in the NCBI data base [62] and the Ensemble genome projects [63]. 54 previously published sequences were collected, representing 26 different species from the main phylogenetic groups of the chordates phylum: two cephalochordates, 10 fish, two amphibians, six reptiles and birds and six mammals. **Table S1** shows the species names and reference numbers for each collected sequence. Two melanopsin sequences from the sea urchin (*Strongylocentrotus sp.*) were included as outgroups. All the sequences from the *InRHO* photoreceptors were retrieved from the Davies *et al.* 2010 [16].

### Sequence Alignments and Phylogenetic Trees

A protein-based coding-sequence alignment was performed with the translated nucleotides sequences and the standard options of the Muscle version-3.3 algorithm [64], which was subsequently improved by manual inspection of the alignment. The quality of the alignment was enhanced with the Gblocks web server [65] by removing ambiguous and gaps-rich sites (>75% gaps). We then used three alignment sets in further analyses: (i) the default settings; (ii) eliminating sites with more than 75% of gaps; and (iii) removing gap-rich sites but considering the codon information (used for the positive selection analyses).

The presence of saturation in base substitution for the *OPN4* and *OPN4m* and *OPN4x* variants was tested by comparing half of the theoretical saturation index expected when assuming full saturation (I_SS.C_, critical value) with the observed saturation index (I_SS_) [66]. No evidence of saturation in any of the referred alignments (**table S4**). jModelTest version 0.1.1 [67] implementing the Akaike Information criterion (AIC) was used to estimate the most appropriate model of nucleotide substitution for tree construction analysis. This procedure was repeated for each melanopsin paralogs genes, with the *OPN4m* and the *OPN4x* sequences. GTR+I+Γ was determined as the best-fit model for *OPN4*, *OPN4m* and *OPN4x* alignments. The estimated parameters under the selected nucleotide substitution model for each gene can be seen in **table S4**.

Phylogenetic trees were constructed using two distinct algorithms, Maximum likelihood (ML) in PhyML [68] and Bayesian analysis in Mr. Bayes 3.1.2 [69], [70], using the estimated parameters found for the nucleotide evolutionary model determined earlier. Bootstrap analyses (1000 replicates) were used to assess the relative robustness of branches of the ML tree [71]. Bayesian analysis was conducted using the estimated parameters of the nucleotide substitution model as priors for 5.000.000 generations. Two concurrent runs were conducted to verify the results. The first 12500 trees were discarded as burn-in samples, the remaining trees were used to compute a majority-rule consensus tree with posterior probabilities. Synteny analyses were performed using the Ensembl and Genomicus version 64.1 data bases [63], [72].

### Positive Selection Assessment

*OPN4*, *OPN4m* and *OPN4x* alignments and the ML/Bayesian trees were used in the program codeml from the PAML version 4.4 software package [30] to assess the selective pressure acting on melanopsin sites. To examine the *dN*/*dS* or ω ratio, three codon substitution models of maximum likelihood analysis were performed: branch-specific, site-specific and branch-site likelihood models.

The site specific models were tested comparatively [73]: M0 (one ratio) *versus* M3 (discrete), M1a (nearly neutral) *vs* M2a (positive selection) and M7 (beta) *vs* M8 (beta+ω). Subsequent likelihood rate comparisons were performed to test which models fits the data significantly better. Model M0 assumed a constant ω-ratio, while in models M1a and M2a ω-ratio is supposed to be variable between sites. M7 and M8 assume a β-distribution for the ω value between 0 and 1. Models M2a, M3 and M8 allow the occurrence of positively selected sites. In addition, the ω value for each codon of the melanopsin *OPN4m* and *OPN4x* paralogs was assessed under the significantly selected site model, using the Selecton web server [74].

The branch selection models were implemented comparing the same ω ratio for all lineages in the tree (one-ratio model) and the two-ratio models assigned two ω ratios for the foreground (ω_1_) and background branches (ω_0_) [75]. The branch-site models allow the ω ratio to vary both among sites and among lineages and were used to detect positive selection that affects only a few sites along a few lineages. A most stringent branch-site test of branch-site test of positive selection was implemented comparing the alternative model A and the ω fixed null model [76]. When the likelihood ratio test was significant, the Bayes Empirical Bayes (BEB) method was used to calculate posterior probabilities of the sites that are subject to positive selection [77].

Branch-specific and branch-site models were implemented to study the melanopsin duplication event and both followed the approach outlined here: model A represents the selective pressure before the duplication event and models B and C had one ω value for each duplicated lineage following the duplication event. The significance for the referred likelihood ratio tests (LRTs) was calculated using the chi-square approximation 2Δ*lnL*, the double of the difference between the alternative and null model log likelihoods. LRT degrees of freedom are calculated as the difference of free parameters between the nested models.

A protein level analysis to detect possible positively selected sites were also investigated on the basis of 31 physicochemical criteria with TreeSAAP version 3.2 [35]. TreeSAAP measures the selective influences on structural and biochemical amino acid properties during cladogenesis, and performs goodness-of-fit and categorical statistical tests. The program classifies the range of changes in eight magnitude categories from conservative to radical for each amino acid properties and calculates a *z*-score that indicates the direction of selection (negative or positive selection) [78]. Positive radical or destabilizing selection sites (6, 7 and 8 magnitudes) as expected to result in significant structural and functional changes on the protein were monitored at the 0.01 significance level.

### Structural Analysis and Homology Modeling

Three-dimensional homology models of melanopsin were built using Modeller version 9.9 [79] implementing a comparative protein structure by satisfying spatial restraints. Squid (*Todarodes pacificus*) rhodopsin protein data bank available structures 2ZIY [80] and 2Z73 [81] were selected as homology models. The predicted three-dimensional conformation of *Gallus gallus* m and x-type melanopsin was based on the invertebrate squid (*Todarodes pacificus*) rhodopsin protein 2ZIY [80]. Consurf webserver was implemented to calculate the conservation index and to assess the three-dimensional localization of most variable and conserved domains at the melanopsin molecule [36]. PyMol version 1.4 graphical interface was used to manipulate the melanopsin molecule and to perform all the images that include melanopsin three-dimensional structure [82].

### Functional Divergence

Diverge version 2.2 was used to identify sites of type I and type II functional divergence, which occurs through changes in the amino acids biochemical properties at a specific positions between defined groups of related proteins [37]. The functional divergence between two monophyletic groups can be classified in two groups: (i) type I, if the amino acid pattern are very conserved in the duplicate gene but highly variable in the other gene copy, which implies shifted functional constrains and (ii) type II, when the amino acid pattern is very conserved in both the duplicated gene clusters but their biochemical properties are very different [83]. Type I and type II functional divergence was assessed by estimating the θ_I_ and θ_II_ divergent coefficients. θ parameter significantly greater than zero means that either altered selective constraints or a radical shift of amino acid physiochemical property after gene duplication is likely to have occurred [38], [84]. A site-specific outline based on the posterior probability (>0.75) was used to predict critical amino acid residues that were responsible for functional divergence between groups. Pred-Couple 2.0 tool was implemented to predicted coupling specificity of GPCRs to the four known G-proteins families [39]. The predicted coupling specificity robustness of the melanopsin sequences was evaluated with the generated posterior probability.

## Supporting Information

Figure S1 **Melanopsin gene tree including the lamprey (** ***Petromyzon marinus*** **) blasted sequence ENSPMAG00000006406.** ML and Bayesian method were performed to build the phylogenetic tree. Bootstrap and posterior probability support values are respectively represented for each node.
(PDF)

Figure S2 **Comparative importance of destabilizing positive selected substitutions in the** ***OPN4m*** **and** ***OPN4x*** **paralogs for each amino acid property.**
(PDF)

Table S1 **Melanopsin sequences used in the phylogenetic analysis.**
(PDF)

Table S2 **Number and relative frequency of the destabilizing positively selected substitutions in the OPN4m and the OPN4x paralogs.** 30 physicochemical properties were analysed in two categories, based on their nature: chemical and structural.
(PDF)

Table S3 **Branch and branch-site selection tests and the respective estimated parameters.** The asterisk (*) means that the alternative hypothesis is statistically significant at a 5% level, implementing the LRT (likelihood ratio test). Notes: *df* – degrees of freedom.
(PDF)

Table S4 **Nucleotide substitution models and the respective estimated parameters for OPN4m, OPN4x and OPN4 alignments.** Parameters: base frequencies, substitution ratio between the nucleotide bases (*r*), gamma shape parameter and proportion of invariable sites (*p*-inv). The comparison between the saturation index (I_SS_) and the critical index value (I_SS.C_) implemented by Xia et al. 2003 [80] were also represented, as well as the respective category of data saturation.
(PDF)
