# Supplementary figures and images for: The Role of Gene Duplication and Unconstrained Selective Pressures in the Melanopsin Gene Family Evolution and Vertebrate Circadian Rhythm Regulation
Source: PLoS One. 2012 Dec 21;7(12):e52413. doi: 10.1371/journal.pone.0052413 (PMC3528684; doi:10.1371/journal.pone.0052413)

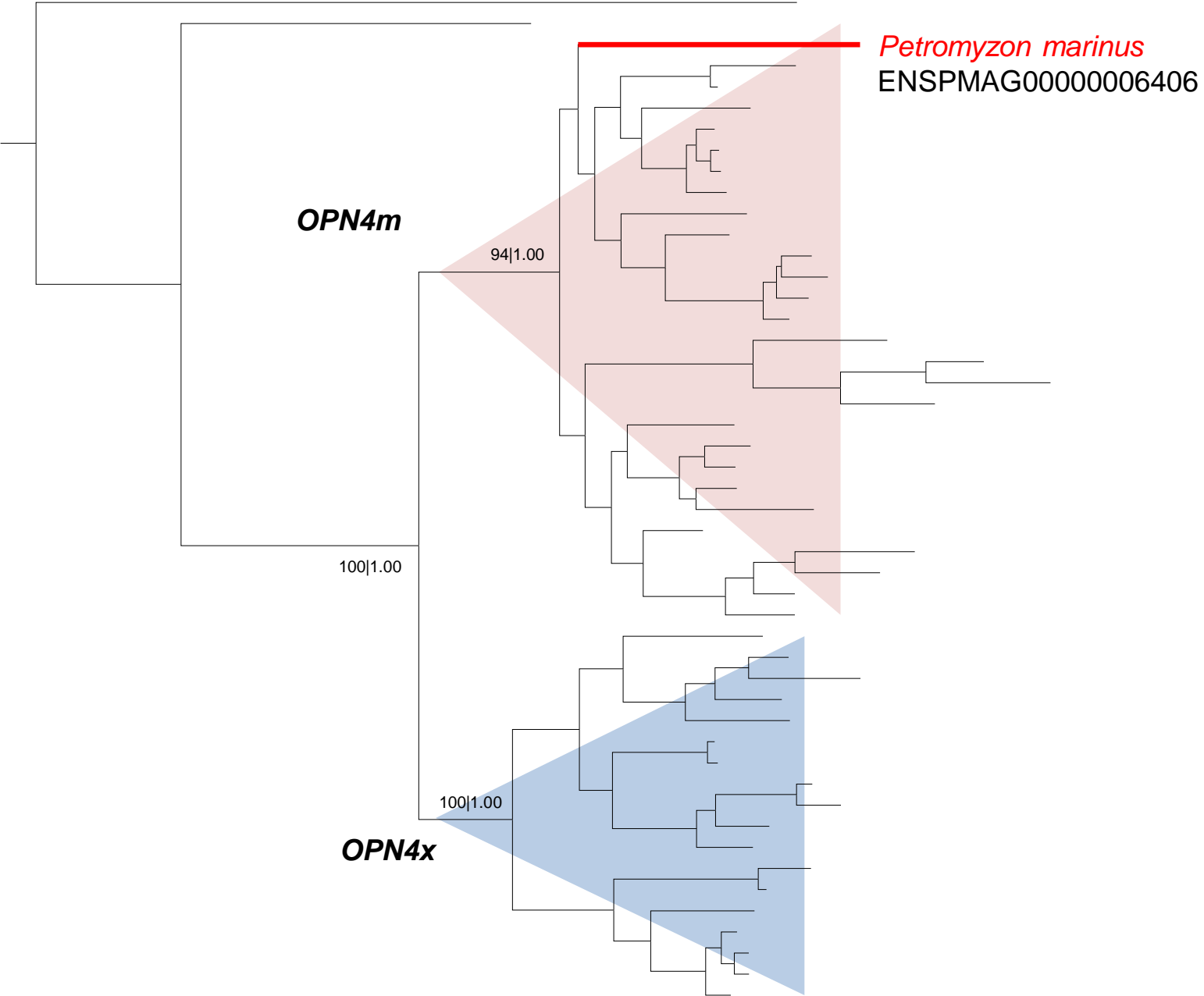

Supplement: Figure S1 — Melanopsin gene tree including the lamprey ( Petromyzon marinus ) blasted sequence ENSPMAG00000006406. ML and Bayesian method were performed to build the phylogenetic tree. Bootstrap and posterior probability support values are respectively represented for each node. (PDF) [file pone.0052413.s001.pdf]

relative frequency of substitutions

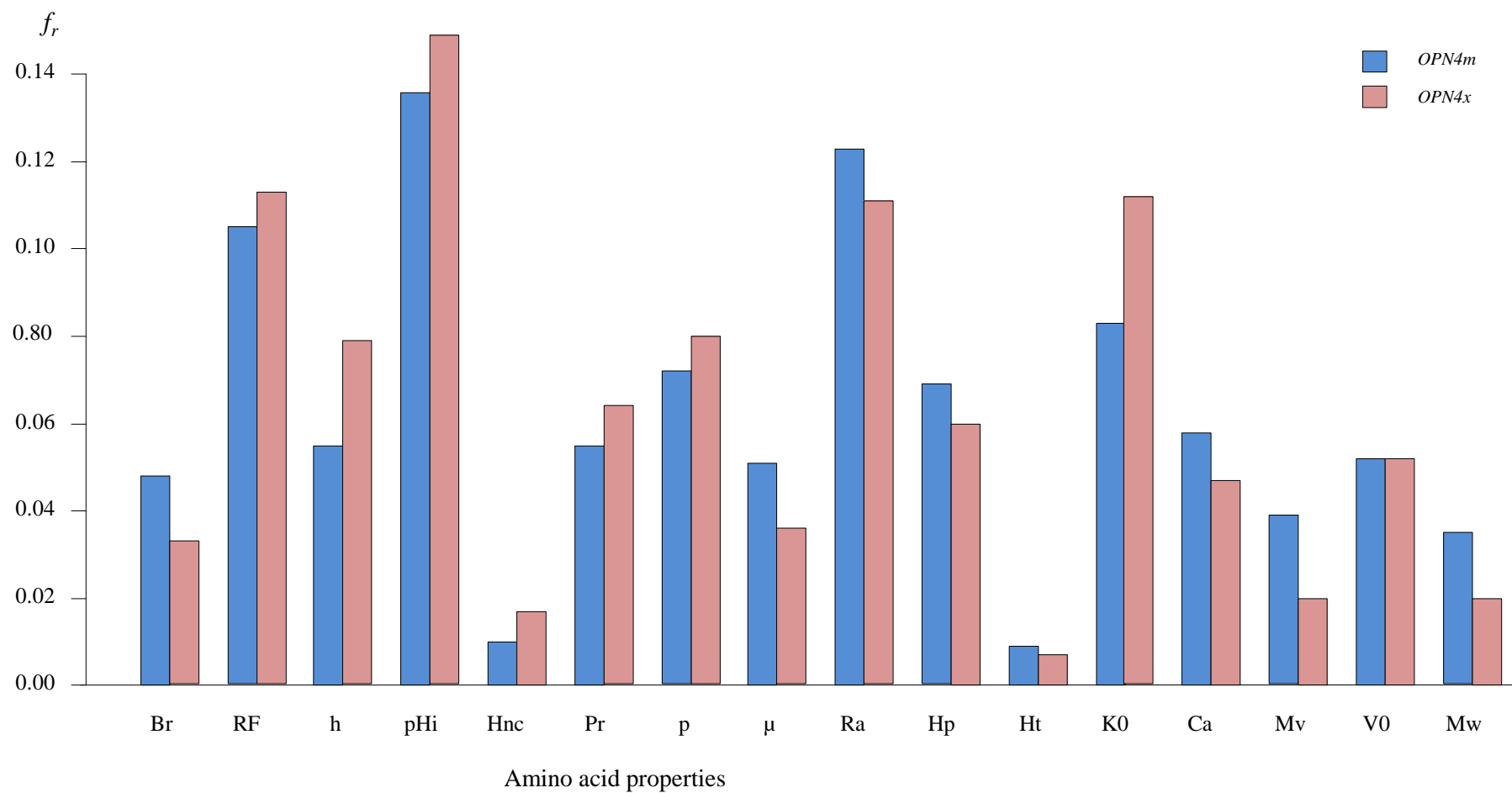

Supplement: Figure S2 — Comparative importance of destabilizing positive selected substitutions in the OPN4m and OPN4x paralogs for each amino acid property. (PDF) [file pone.0052413.s002.pdf]
