# Supplementary material for: The Role of Gene Duplication and Unconstrained Selective Pressures in the Melanopsin Gene Family Evolution and Vertebrate Circadian Rhythm Regulation
Source: PLoS One. 2012 Dec 21;7(12):e52413. doi: 10.1371/journal.pone.0052413 (PMC3528684; doi:10.1371/journal.pone.0052413)
Supplement: Table S1 — Melanopsin sequences used in the phylogenetic analysis. (PDF) [file pone.0052413.s003.pdf]

Specie	Name	NCBI/ENSEMBLE	Paralog
Strongylocentrotus droebachiensis	S. droebachiensis	DQ285097.1	.
Strongylocentrotus purpuratus	S. purpuratus	XM_779466.2	.
Branchiostoma floridae	B. floridae	XM_002596237.1	.
Branchiostoma belcherii	B. belcherii	AB205400.1	.
Danio rerio	D. rerio 1	ENSDARG00000051908	*x*
	D. rerio 2	ENSDART00000108727	*x*
	D. rerio 5	ENSDARG00000007553	*m*
	D. rerio 6	XM_689400.2	*m*
Takifugu rubripes	T. rubripes 1	ENSTRUG00000013541	*x*
	T. rubripes 2	ENSTRUT00000015619	*x*
	T. rubripes 3	ENSTRUG00000011365	*m*
	T. rubripes 4	ENSTRUG00000000307	*m*
Gasterosteus aculeatus	G. aculeatus 1	ENSGACG00000016885	*x*
	G. aculeatus 2	ENSGACG00000007757	*x*
	G. aculeatus 4	ENSGACG00000011528	*m*
	T. nigroviridis 2	ENSTNIG00000009056	*x*
	T. nigroviridis 3	ENSTNIG00000003074	*m*
	T. nigroviridis 4	ENSTNIG00000007169	*m*
Oryzias latipes	O. latipes 1	ENSORLG00000005512	*x*
	O. latipes 2	ENSORLG00000007864	*x*
	O. latipes 3	ENSORLG00000007679	*m*
	O. latipes 4	ENSORLG00000010483	*m*
Ictalurus punctatus	I. punctatus 1	NM_001200310.1	*m*
	I. punctatus 2	FJ839437.1	*m*
Gadus morhua	G. morhua1	AF385823.1	*x*
	G. morhua2	AY126448.1	*x*
Coryphaenoides armatus	C. armatus 1	EU479707.1	*x*
Astatotilapia burtoni	A. burtoni	EU523855.1	*m*
Rutilus rutilus	R. rutilus	AY226847.1	*m*
Xenopus laevis	X. laevis 1	AF014797.1	*x*
Anolis carolinensis	A. carolinensis 1	XM_003221173.1	*x*
Podarcis siculus	P. siculus	DQ013043.2	*x*
Gallus gallus	G. gallus 1	ENSGALG00000010408	*x*
	G. gallus 2	ENSGALG00000001934	*m*
Meleagris galopavo	M. galopavo 1	ENSMGAG00000005644	*x*
	M. galopavo 2	ENSMGAG00000002543	*m*
Anas platyrhynchos	A. platyrhynchos 1	ENSAPLG00000004330	*x*
	A. platyrhynchos 2	ENSAPLG00000014594	*m*
Taeniopygia guttata	T. guttata 1	ENSTGUG00000003147	*x*
	T. guttata 2	ENSTGUG00000005687	*m*
Sminthopsis crassicaudata	S. crassicaudata	DQ383281.1	*m*
Monodelphis domestica	M. domestica	ENSMODG00000011114	*m*
Homo sapiens	H. sapiens	ENSG00000122375	*m*
Canis familiaris	C. familiaris	ENSCAFG00000015975	*m*
Loxodonta africana	L. africana	ENSLAFG00000006004	*m*
Rattus norvegicus	R. norvegicus	ENSRNOG00000011600	*m*
