# Supplementary material for: The Role of Gene Duplication and Unconstrained Selective Pressures in the Melanopsin Gene Family Evolution and Vertebrate Circadian Rhythm Regulation
Source: PLoS One. 2012 Dec 21;7(12):e52413. doi: 10.1371/journal.pone.0052413 (PMC3528684; doi:10.1371/journal.pone.0052413)
Supplement: Table S2 — Number and relative frequency of the destabilizing positively selected substitutions in the OPN4m and the OPN4x paralogs. 30 physicochemical properties were analysed in two categories, based on their nature: chemical and structural. (PDF) [file pone.0052413.s004.pdf]

		OPN4m			*OPN4x*		
Amino acid properties		Number of positive destabilizing	f_r		Number of positive destabilizing	f_r	
Bulkiness	B_r	58	0.048		36	0.033	
Chromatographic index	R_F	127	0.105		121	0.113	
Hydropathy	h	67	0.055		85	0.079	
Isoelectric point	pH_i	165	0.136		160	0.149	
Normalized consensus hydrophobicity	H_{nc}	12	0.010		18	0.017	
Polar requirement	P_r	67	0.055		69	0.064	
Polarity	p	87	0.072		86	0.080	
Refractive index	μ	62	0.051		39	0.036	
Solvent accessible reduction ratio	R_a	150	0.123		119	0.111	
Surrounding hydrophobicity	H_p	84	0.069	Chemical	65	0.060	Chemical
Thermodynamic transfer hydrohphobicity	H_t	11	0.009	0.733	8	0.007	0.750
Compressibility	K^0	101	0.083		120	0.112	
Helical contact area	C_a	71	0.058		50	0.047	
Molecular volume	M_v	47	0.039		22	0.020	
Partial specific volume	V^0	63	0.052	Structural	56	0.052	Structural
Molecular weight	M_w	43	0.035	0.267	21	0.020	0.250
**Total**		1215			1075		

* 15 chemical and 15 structural properties were analysed but only the represented give signficant results.

Legend:

f_r relative frequency of substituitions for each propertie
