# Supplementary material for: The Role of Gene Duplication and Unconstrained Selective Pressures in the Melanopsin Gene Family Evolution and Vertebrate Circadian Rhythm Regulation
Source: PLoS One. 2012 Dec 21;7(12):e52413. doi: 10.1371/journal.pone.0052413 (PMC3528684; doi:10.1371/journal.pone.0052413)

Branch models		Likelihood	*p*	ω	LRT	*df*	
OPN4m /*OPN4x*							
Model O		-20998.594	92	0.077			
Model A		-20998.323	93	0.006	0.541	1	
Model B	*OPN4x*	-20992.982	93	∞	11.223	1	*
Model C	*OPN4m*	-20994.886	93	∞	7.416	1	*
OPN4m3 /*OPN4ma*							
Model O		-12319.188	46	0.089			
Model A		-12317.165	47	∞	4.046	1	*
Model B	*OPN4m3*	-12317.518	47	0.209	3.338	1	
Model C	*OPN4ma*	-12318.228	47	0.296	1.920	1	
OPN4x1 /*OPN4x2*							
Model O		-10568.326	38	0.089			
Model A		-10568.035	39	0.063	0.582	1	
Model B	*OPN4x2*	-10566.161	39	∞	4.330	1	*
Model C	*OPN4x1*	-10566.122	39	0.902	4.408	1	*

Branch site models		Likelihood	*p*	ω	*p+*	LRT	*df*	
OPN4m /*OPN4x*								
Model O		-20819.380	94					
Model A		-20814.236	95	159.543	0.168	10.289	1	*
Model B	*OPN4x*	-20814.352	95	∞	0.248	10.056	1	*
Model C	*OPN4m*	-20817.482	95	∞	0.183	3.794	1	
OPN4m3 /*OPN4ma*								
Model O		-12108.048	48					
Model A		-12103.212	49	667.161	0.027	9.673	1	*
Model B	*OPN4m3*	-12088.628	49	593.875	0.110	38.841	1	*
Model C	*OPN4ma*	-12107.509	49	13.128	0.019	1.079	1	
OPN4x1 /*OPN4x2*								
Model O		-10461.138	40					
Model A		-10461.138	41	1.000	0.087	0.000	1	
Model B	*OPN4x2*	-10464.285	41	∞	0.032	6.294	1	*
Model C	*OPN4x1*	-10458.370	41	33.942	0.050	5.537	1	*

Legend

p	number of parameters
ω	omega-value
LRT	likelihood ratio test
df	degrees of freedom
p+	proportion of positively selected sites

Models

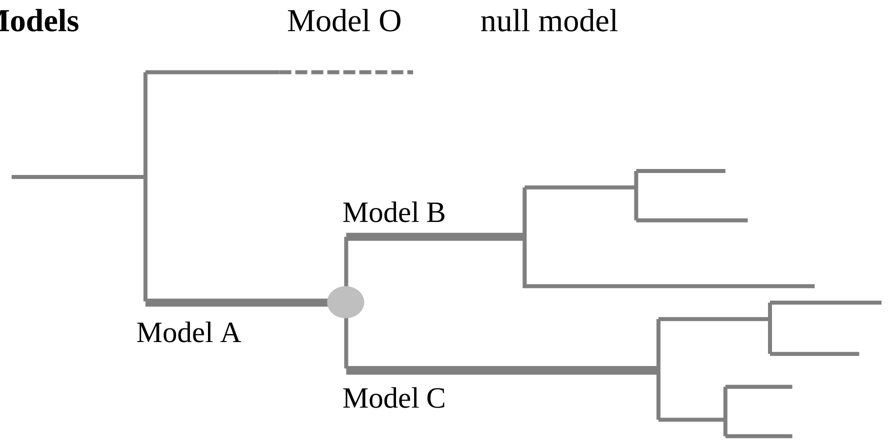

Supplement: Table S3 — Branch and branch-site selection tests and the respective estimated parameters. The asterisk (*) means that the alternative hypothesis is statistically significant at a 5% level, implementing the LRT (likelihood ratio test). Notes: df – degrees of freedom. (PDF) [file pone.0052413.s005.pdf]
