# Supplementary material for: The Role of Gene Duplication and Unconstrained Selective Pressures in the Melanopsin Gene Family Evolution and Vertebrate Circadian Rhythm Regulation
Source: PLoS One. 2012 Dec 21;7(12):e52413. doi: 10.1371/journal.pone.0052413 (PMC3528684; doi:10.1371/journal.pone.0052413)
Supplement: Table S4 — Nucleotide substitution models and the respective estimated parameters for OPN4m, OPN4x and OPN4 alignments. Parameters: base frequencies, substitution ratio between the nucleotide bases (r), gamma shape parameter and proportion of invariable sites (p-inv). The comparison between the saturation index (ISS) and the critical index value (ISS.C) implemented by Xia et al. 2003 [80] were also represented, as well as the respective category of data saturation. (PDF) [file pone.0052413.s006.pdf]

Nucleotide substitution model

	OPN4m	***OPN4x***	***OPN4***
Model	GTR+I+G	GTR+I+G	GTR+I+G
A	0.179	0.233	0.197
C	0.337	0.285	0.314
G	0.267	0.234	0.255
T	0.218	0.249	0.234
r [*AC*]	1.117	1.619	1.374
r [*AG*]	3.663	3.822	3.724
r [*AT*]	1.359	1.137	1.355
r [*CG*]	0.884	1.290	0.924
r [*CT*]	3.271	3.364	3.337
r [*GT*]	1.000	1.000	1.000
p-inv	0.090	0.246	0.227
gamma shape	1.430	1.242	0.745

Xia et al. 2003 saturation test

	OPN4m	***OPN4x***	***OPN4***
ISS	0.385	0.366	0.473
ISS.c	0.767	0.767	0.775
t -test	18.088	19.826	14.504
df	991	1022	885
p -value	0.000	0.000	0.000
	Little saturation	Little saturation	Little saturation
